# Adolescents' Perceptions of Their Consent to Psychiatric Mental Health Treatment

**DOI:** 10.1155/2012/379756

**Published:** 2012-02-16

**Authors:** Anthony James Roberson, Diane K. Kjervik

**Affiliations:** ^1^Capstone College of Nursing, The University of AL, Tuscaloosa, Alabama 35487-0358, USA; ^2^Health Care Environments Division, School of Nursing, University of North Carolina at Chapel Hill, 528 Carrington Hall, Chapel Hill, NC 27599-7460, USA

## Abstract

The purpose of this paper is to present the findings of a small-scale study in which the decision-making process of adolescents who consent to psychiatric mental health treatment was examined. Sixteen (16) adolescents were interviewed about their decisions related to initial and continued treatment, along with their understanding of minor consent laws. Interviews were audio-recorded, and transcripts were analyzed through concept analysis. Findings are presented in the context of the decision-making steps and research questions. Most adolescents did not recognize consequences related to psychiatric mental health treatment and did not assimilate and integrate information provided to them about treatment choices. Adolescents disagreed with current minor consent laws that allow minors to consent to certain healthcare treatments without the required consent of the parent. Further, adolescents reported that a collaborative approach in making decisions about the adolescent's psychiatric mental health treatment was most facilitative of achieving the goals of treatment.

## 1. Introduction

Adolescence is uniquely different from all other stages of human development, especially from physiological and cognitive perspectives, and it can be argued that it is the most challenging of all developmental periods [1]. The physical changes that occur during this developmental stage are perhaps more obvious than the cognitive changes. The adolescent experiences genital development, breast development, pubic and axillary hair development, skin changes, and at times rapid changes in height and weight [1]. The physiological changes of development will be realized eventually for each adolescent. Further, physiological changes generally occur earlier in girls than boys.

The cognitive changes of adolescents occur with great diversity. “Many adolescents are as egocentric in some respects as preschool children, while others reach the stage of abstract thinking that characterizes advanced cognition” [1, page 351]. Adolescence is the period of development that the individual is usually attempting to break parental bonds, establish themselves in certain social groups, and develop a sense of self [2].

In addition to establishing one's identity, the adolescent is also striving to become more independent. Adolescence is perhaps the phase of development in which the individual is making the most effort to seek independence and control over their lives, which includes the desire to start making more of their own decisions [3, 4]. Piaget defines the cognitive developmental stage of formal operational thinking as the phase in which adolescents aged 12 years and older can think about hypothetical concepts and are able to contemplate consequences related to decision choices [1]. Part of the cognitive development of adolescents aged 12 years and older includes the adolescent's increased ability to solve problems and “speculate about the possible as well as the real” more independent of others [1, page 56].

One area of decision-making research that has recently been examined more closely is that of adolescents making independent decisions about their healthcare treatment [5–7]. These researchers suggest that adolescents are capable of making complex healthcare decisions. However, adolescents have shown that they are more interested in having their developmental needs, such as independence and autonomy, met and at times may forgo the recommended treatment in order to meet these needs [8].

Research studies support that parents and peers influence the decision-making of adolescents in hypothetical healthcare situations [9–11]. Parents are more influential than peers when the adolescent is making a decision that has a moral or value slant, such as deciding to report someone who has destroyed property or has engaged in stealing. Peers are more influential when the adolescent is deciding on non-life-threatening issues, such as whom to date and what to wear to an event. However, the influence that parents and peers have on the adolescent who is deciding to consent in a real life healthcare situation is not fully understood. Further, we do not understand the influences of parents, family, and peers on outcomes in psychiatric mental health situations.

The informed consent rights of minors have been expanded in recent years [12, 13]. English and Kenney [13] have provided a comprehensive monograph describing each state's minor consent laws related to healthcare treatments. Although these laws vary in the types of healthcare treatments, the adolescent may consent, most states allow minors to consent to certain medical procedures, such as mental health services, including outpatient and crisis intervention for mental health reasons, without the required consent of the parent or legal guardian [13].

The purpose of this paper is to provide the findings of a descriptive qualitative study in which the decision-making process of adolescents who consent to psychiatric mental health treatment in real life situations was examined. Considering the minor consent laws that provide adolescents the right to consent to psychiatric mental health treatment without the consent of the parent, the following research questions were addressed in the study.

1. How do 12-to-17-year-old adolescents who consent to psychiatric mental health treatment (medication intervention, psychotherapy, or a combination of both) perceive the process of deciding to accept treatment?
2. How do 12-to-17-year-old adolescents who consent to psychiatric mental health treatment (medication intervention, psychotherapy, or a combination of both) perceive the goals of treatment?

## 2. Methods

### 2.1. Subjects

Sample size in qualitative research is determined by theoretical data saturation, rather than power analysis, which is associated with quantitative research [14]. The sample size for this study consisted of 16 adolescents. The age range of the adolescents was 12 to 17 years (nine 12 to 14 years old and seven 15 to 17 years old). These age groups represent the major adolescent developmental stages. The study included a relatively equal number of females (9) and males (7). The intent for this inclusion was to identify any differences between genders in problem-solving abilities. The study included 10 African American and 6 Caucasian adolescents.

An outline of the demographic information for the adolescent and parent participants is provided in Tables 1, 2, 3, and 4.

Full approval for this research was obtained from the University of North Carolina at Chapel Hill Public Health and Nursing Institutional Review Board. Adolescents whose only language was English were recruited and included in this study. All adolescents who participated in this study were from lower socioeconomic families. The sample was purposeful in that adolescents recruited for the study had consented to treatment and were receiving out-patient psychiatric mental health treatment. Psychiatric mental health treatment is defined as a combination of medication and psychotherapy intervention.

Only adolescents who were receiving maintenance psychiatric mental health treatment were considered for this study. Maintenance treatment was defined by the American Psychiatric Association [15] criteria as a period of treatment when the patient is not experiencing recurrent signs and symptoms of the illness being treated. Although *maintenance phase* is not defined by the number of weeks or months, the criteria established for this study were as follows: over the past four weeks, the adolescent's medications had not been changed or adjusted either in dosing or type, and new issues in therapy were not being addressed.

### 2.2. Setting

Data collection took place in a community-based out-patient psychiatric mental health practice located in a mid-sized city in the Southeast. Services provided by the facility include medication intervention, individual psychotherapy, group therapy, and case management services. Psychiatric Mental Health Nurse Practitioners provide medication management in addition to individual and family therapy for patients. Master's and doctoral level therapists provide individual and group therapy. Case management is provided by bachelor's prepared therapists.

### 2.3. Procedure

After the appropriate informed consents were obtained by this researcher, adolescents were interviewed using a semistructured interview script [16]. Although the intent was to consistently follow the interview script [16], participants were encouraged to discuss other matters not included in the interview questions that were related to the research topic. The final interview script was pilot-tested with three adolescents to ensure that the interview questions could be understood and comprehended. The adolescents who participated in the pilot test were not included in the final data analysis of this study.

Interviews with the adolescent were audio-recorded with the use of an Olympus digital recorder and took place in a private, sound-proof room at the study facility. Each interview lasted for approximately one hour. Specific consent for this author to read the medical records of the adolescents was obtained. The information extracted from the medical records included demographic data about the adolescent, such as year of birth, ethnicity, mental health diagnosis, medical diagnosis, and education level.

### 2.4. Data Management and Analysis

Data analysis was a continuous process throughout the study [17]. The researcher transcribed the data after each interview. All field notes were transcribed by the principal investigator (PI). Field notes contained a detailed description of the events that occurred during the interview, such as the location, date, and time of the interviews and any events that were observed during the interview. These notes also included what was learned by this author from one interview to the next. The field notes were organized chronologically in a loose-leaf binder [14].

Each participant was assigned a number that was only identifiable by this researcher. Each document that included data (transcripts from adolescent, demographic information, field notes) was assigned the participant number. No participant-identifying information was linked to the transcripts, audiotapes, field notes, or demographic information. All collected data were categorized according to date and time of interview, and the deidentifying number assigned to each participant.

The data analysis process was completed using several components. Verbatim transcripts were read iteratively [18]. A system of data reduction, through open, *in vivo*, and axial coding [19] techniques was used when appropriate. Members of the research team and psychiatric mental health therapists and nurse practitioners assisted in evaluating the coding schemes. The components and subcomponents of coded data were analyzed to develop themes, and then the themes were related to the research questions and synthesizing framework [18, 20, 21]. Collected field notes were used to enhance the insights provided by the transcripts [18].

Content analysis [22] of the data was completed for this study. This approach was helpful considering the limited knowledge on the topic of the decisions adolescents make in psychiatric mental health situations [22, 23]. Data were categorized, in this case according the interview questions [23]. The categories were further separated into indexes and are represented by exemplaries included as support for the findings. The units that were analyzed in this study were primarily phrases and sentences from the adolescent interviews.

Validation of data was crucial to ensure the accuracy of the data and reliability of the findings. To make certain, validity was optimized, accuracy of the transcripts was thoroughly scrutinized by this researcher. Creswell and Miller [24] state that validity in qualitative studies is defined by how accurately the account of the study participants' realities is presented in the data analysis. The researcher was most concerned with the inferences drawn from reported study results and whether the realities of the participants were provided. Therefore, validity of the findings was checked by asking the study participants themselves if the interpretations by the PI truly captured the essence of their experiences [18]. Another test of validity involved the PI sharing the descriptions and the steps of analysis with qualitative experts [14, 18].

## 3. Results

The following decision-making step model was used as the framework in formulating the interview questions and will serve as a guide to present the findings: (a) the adolescent's recognition that a treatment decision is required, (b) the adolescent's understanding of treatment goals, (c) the ability of the adolescent to determine the consequences of the treatment decisions, and (d) the adolescent's ability to understand that each consequence is likely to occur, which includes assimilating and integrating the information provided about treatment options [25–27]. In addition to the decision-making model, the study findings were also presented in terms of the research questions.

## 4. Research Question No. 1: Adolescents' Perceptions of Their Decision-Making Process about Treatment

### 4.1. Recognizing That a Treatment Decision Was Required

Adolescents were first asked about the decisions they made in initiating psychiatric mental health treatment with the following: “tell me about the initial decisions you made about getting treatment” and “how did you decide that you needed treatment?” The researcher was specifically interested in how the adolescents came to the decision that they were in need of treatment. Particular attention was given to those decisions that the adolescent made without the parent or legal guardian to initiate treatment. It was important to distinguish between those decisions that the adolescent made without others and those made with others in order to fully understand if the adolescent recognized that a decision about their mental health treatment was required.

*“I knew I needed to get some help, I had to.” *(John, 17-year-old male) All adolescents reported that during the initial meeting with the nurse practitioner or therapist, they realized that a decision about treatment choices would be needed. Most (*n* = 12) adolescents stated that the initial decision to seek treatment was completed in collaboration with their parent or legal guardian. Some adolescents (*n* = 4) reported that decisions made about initiating treatment were made without the parent. All adolescents felt their own input was taken seriously by their parent and nurse practitioner, whether in making independent decisions or decisions in conjunction with their parent or legal guardian.

There was strong evidence to support the adolescent's ability to identify that a decision about treatment was needed or ultimately required. For example, a common response among adolescents was that they knew when the initial appointment was made at the treating facility; there would ultimately be a decision made about accepting or rejecting treatment. All adolescents identified that the general purpose of the initial visit to the facility was to receive psychiatric mental health treatment and that a decision about their treatment would be required. Although all adolescents engaged in discussions about their initial decisions to seek treatment, the older adolescents (15 to17 years old) provided more detailed and focused descriptions regarding their perceptions of need for treatment.

The interview questions included asking adolescents about the initial treatment-related decisions. Each adolescent was asked about the purpose of seeking treatment and more specifically about starting treatment. The purpose of posing questions in this manner was to determine exactly what decisions the adolescents made about their treatment. It is evident that all adolescents could recognize that a decision to seek mental healthcare was needed, based on their negative experiences behind and ahead of them:

*I was on drugs real bad and I started, when I, I felt real bad one day. I was like crying all of the sudden, I donot know. I had this feeling that something werenot right so I went to [private hospital]. I told my mom that I wasnot feeling great, so she took me to the hospital and I was the one who wanted to go but she kind of helped me out with that. Then I wanted to come here because [private hospital] didnot think I needed to be in the hospital. So, I knew I needed to get some help, I had to. *(John, 17-year-old male)

**Understanding the Goals of Treatment**

Adolescents were asked specific questions related to their goals of treatment to understand whether the adolescent could identify personal treatment goals, rather than those established by others (i.e., nurse practitioner, therapist, parents). Each adolescent voiced specific goals for treatment, including those independently formulated and those that seemed influenced by others.

*“I just wanted to feel better.” *Most adolescents based their goals of treatment on the struggles experienced in living with their mental illness. One 17-year-old male diagnosed with a major depressive disorder, severe type, communicated that his goal in treatment was to “feel better and to not be so depressed…I was not doing well in school because of the depression and I needed to get my grades better…but I had to get over the depression first.” Other examples of goals were

*I wanted to, uh, stop stressing all the time and stop fighting and stuff and fighting to [sic] my little sister. *(Lauren, 15-year-old female)

*I wanted to be able to control myself better and bring my grades up…I was feeling real bad and I just wanted to feel better. *(John, 17-year-old male)

*“To not end up like my mother.”* It was evident in some responses (*n* = 3) that some adolescents may have formulated an answer about their goals in treatment based on what they had been told by others. For example, a 12-year-old male who had been sexually molested by his mother for many years reported his goal in treatment was “to not end up like my mother because she molested us [siblings] when we were little and I do not want to molest my kids when I get older.” The concept that certain behaviors, such as molestation, are somehow “passed down” is at a higher level of logic and abstraction that would normally be expected of an older adolescent, but not of a 12-year-old's thought pattern. This response suggests that an adult discussed this particular goal with him. Regardless of his age, this adolescent was capable of stating a treatment goal and he had learned from prior experiences.

*“To feel better now.” *Initially, questions about their short-term goals and responses were highly detailed. However, when questioned about any long-term goals that were established in the initial stages of treatment, adolescents would consistently refer back to the short-term goals of feeling better now, improving grades, or to stop being angry. The adolescents were unsuccessful in identifying long-term goals established at the onset of their treatment. But, they were successful in discussing immediate goals such as to feel better now.

**Determining the Consequences of Treatment Decisions**

To understand whether adolescents could determine the consequences and risks associated with taking medication or engaging in individual or group psychotherapy, the participants were asked to identify the reasons for taking the medications or receiving psychotherapy.

*“The medication helps me calm down.” *Identifying the reasons for any treatment is an important step to being able to identify the consequences. All adolescents spoke easily about their reasons for taking medication. Each rationale was linked with past behavioral experiences that were perceived as negative with positive medication effects. Each related to a context important to the adolescent school performance, emotions, and relationships. The extent of knowledge about the specific reasons for the medications and the elaboration provided by the participants varied, but not by a specific age group. The following are examples of responses when the adolescents were asked to identify the reasons for taking the medications:

*I take Metadate so, it help keep me calmed down a little bit and help me in school…I do better in school, my grades are better…it were given to me because I were hyper a lot, and were gettin' in trouble at school a lot. *(James, 12-year-old male)

*I know the medicine, it [Concerta] helps me concentrate more. *(Sue, 14 year-old female)

*“Wellbutrin influences dopamine levels.” *One 16-year-old adolescent provided a more technical response as to the reason and purpose of the medications he was prescribed. His response differed from other adolescents because he made no mention of how the medication helped him feel better or for what purpose the medication was intended. Even with the use of probes, the adolescent did not seem to make the connection between the goal to feel better and taking an antidepressant. Discussion by the adolescent about the intended purpose and reason for the medication remained at a medical terminology level:

*Um, Wellbutrin, um, influences your dopamine levels, while Lexparo is an SSRI,which focuses more on serotonin, but it does, I think, influence overall levels…like all three brain chemicals. *(Tim, 16-year-old male)

This adolescent's father is a healthcare professional and frequently discusses with his son the reasons for the medications, perhaps on a more technical level than an emotional one.

*“Therapy can relax you.” *Adolescents were also capable of identifying reasons for receiving therapy:

*He's [therapist] teaching me like, um, stuff like tell me do not do bad stuff and what not do and what is good to do. Like he told me I got, um gotta start talking and stuff about stuff that could help me. *(Lauren, 15-year-old female)

*“Antidepressants increase risk for suicide.” *In terms of adolescents identifying consequences and risks associated with medication or psychotherapy intervention, the responses varied according to age. Each adolescent understood the meaning of *risk *as they were asked to provide a definition of the word and an example of “taking a risk”. For example, one adolescent (13-year-old male) defined “taking a risk” as “taking a chance”. He further provided an example by describing, “if you drink and drive, that's a risk of getting a ticket for driving drunk, or it's a risk of hitting someone while you're drunk…having a car accident.” Out of the 11 adolescents taking medications, only one (16-year-old male) provided a clear understanding of the risks involved with taking the medications he was prescribed. This was also the adolescent *(Tim, 16-year-old male)* who discussed the goals of the medication in more technical terms:

*Um, Lexapro, like it has a side effect of, like, like tiredness, which at the beginning I felt a lot, like collapsing in the middle of class until we the times I given [sic] it were switched. I know that Wellbutrin can, is like, has a real risk for seizures…so you always have to be careful about your dose. I know that all antidepressants, especially for adolescents, can increase, like, risk of suicide, well for all ages, but particularly focusing on adolescents because you suddenly have that energy to do things, while it's (antidepressant) not necessarily treating your behavior yet. *(Tim, 16-year-old male)

*“There are no risks.” *Tim's identification of risks associated with taking medications was not typical. In fact, a common theme among the adolescent participants was their inability to identify risks associated with taking the medications they were prescribed. Out of the remaining ten adolescents taking medications, eight stated the medication(s) they were taking had no risks. Two adolescents responded with “I did not know” when asked if the medication(s) they were taking had any risks. The examples reflect that the notion of no risks may be related to the adolescent's perception that they had not experienced any side effects or adverse events, thus there must not be any risks involved in taking the medication:

*No, there ain't no risks…it [Concerta] hadn't done anything wrong to me for the past two years that I been taking it. *(James, 12-year-old male)

*Nothing really…I do not remember what the risks was if there was any…cause I guess they really weren't that bad even if there was em [risks]. I do not know of any [risks]. *(Angela, 15-year-old-female)

One 15-year-old female adolescent identified a side effect of taking Zoloft by stating, “I think I've got to eat with it [Zoloft] so I won't get sick.” One adolescent provided the following response to the risk associated with her medication. Similar responses were shared by others (*n* = 6), but this was the only response this particular adolescent provided in explaining the risks associated with taking Zoloft:

*If I take, if I take a lot of them I know it can do, make an overdose. *(Sue, 14-year-old female)

*“If therapy is helping, how could it hurt?”* No adolescent receiving psychotherapy was able to provide a description of any risks involved in this form of treatment. Each adolescent was asked why they did not think there were any risks associated with receiving therapy. The overwhelming response was similar to “if therapy is helping, how could it hurt?”

In summary, most adolescents did not identify risks associated with taking certain psychotropic medications or receiving psychotherapy. No adolescent identified risks associated with receiving therapy. However, in the adolescent's eyes, the positive effects of improving sadness, providing increased ability to focus, and improving energy levels, satisfied their goals without any thought or consideration to risks or consequences.

**Understanding That Each Consequence Is Likely to Occur**

This step of the decision-making process involves the adolescent assimilating all of the treatment options presented and deciding on the desirability of each consequence. Each adolescent involved in this study was receiving medication, psychotherapy, or a combination of both. To obtain information about the process of assimilation, adolescents were asked questions about their initial treatment decisions in addition to those decisions made about continued treatment. The information gleaned from this interview approach provided an understanding of the process the adolescent went through to incorporate the psychiatric mental health treatment information presented.

## 5. Initial Treatment Decisions

*“We made them together.”* Most (*n* = 12) adolescents perceived that they made the initial decisions about treatment with their parents. Adolescents spoke of the collaboration with their parents in the initial phase of treatment. This collaboration consisted of discussions about whether or not to start medication or psychotherapy intervention:

*We decided that I was going to get treatment and that I would take the medicine that I'm taking. *(Joe, 13-year-old male)

*She [grandmother] wanted me to take the medication and I wanted to take it too, so we decided that together. *(Mack, 15-year-old male)

*“I made the choice.”* Other (*n* = 4) adolescents perceived that they were the ones who decided upon initial treatment, including what kinds of interventions they would consider:

*I'm the one that told them [parents] that I wanted to go [to treatment] andEverything*. (Jim, 17-year-old male)

*I made the choice about me getting into group therapy. *(Jill, 13-year-old female)

## 6. Research Question No. 2: Adolescents' Perceptions of the Goals of Treatment

### 6.1. Continued Treatment Decisions

*“We made those together too.” *The perception among all adolescents was that most decisions made related to continued treatment (those made after the initial treatment decisions) were made with their parents. Examples of continued treatment decisions included goals related to making changes in medications, either in type, dosing levels or timing, or discontinuing therapy:

*Um, I was asked if I wanted to be switched to a different dose and me and my parents talked about that to see if I needed. *(Tim, 16-year-old male)

*My mother asked me if I wanted to stay here [treatment facility], because she would have take me somewhere else if I really wanted to, because I wanted to go to therapy. *(Jill, 13-year-old female)

*“I decided on the mentor I wanted.” *Some (*n* = 6) adolescents reported making some decisions about continued treatment without their parents. These decisions included those made about staying on medications, choosing a mentor, or getting to the appointments at the facility:

*I think the biggest choice I made [about treatment] was not making a big deal out of it, I just kind of went along, but the fact that I just stayed on it [medication], that was not really influenced by my parents. *(Tim, 16-year-old male)

*I decided if I'm feeling something, to just tell my therapist and not hold back like I usually do. *(Barbara, 14-year-old female)

## 7. Adequacy of Adolescent Treatment Decisions

Given the significant decisions made about their treatment, it was important to understand the adolescents' perceptions about the best decisions that were made in treatment. Adolescents were asked “which decisions about your continued treatment do you consider the best; those made without your parent, by your parents without you, or with you and your parents?” “Best” was defined as those decisions that have most led to the adolescent's current psychiatric stability.

### 7.1. The Best Treatment Decisions

The overwhelming theme was that the adolescents (*n* = 16) perceived that the best decisions made about their continued treatment were the ones made in collaboration with their parents. When asked about why they perceived that the best decisions about treatment have been made with their parents, a common response was that collaboration with parents provided them with the opportunity to reflect on the information about treatment more effectively and that some of the treatment decisions could not have been made without their parent. The time spent to mull over the information with their parents prior to deciding was described as important. The discussions with parents about treatment were viewed as helpful in the process and supportive for decision making.

*“The decision me and my grandmom have made” *(Sue, 14-year-old female). Among those (*n* = 11) taking medications, most (*n* = 8) relayed that they would not have been able to make a decision about what type of medication to agree to if this decision would have been made independent of their parent(s)/legal guardian. Although some adolescents (*n* = 2) taking medications relayed that initial decisions related to their treatment were made primarily by their parents, these adolescents perceived that their parents played an integral role in the decisions made about the adolescent's continued treatment.

## 8. Parental Influence from the Adolescent Perspective

When adolescents were asked if parent(s)/guardians were influential in their remaining on the prescribed medication(s) and remaining in therapy, the adolescents' comments indicated the important roles of parents in facilitating treatment through a variety of mechanisms. The adolescents' description of these roles is represented by the following labels: *encourager*, *transporter*, *administrator, *and *purchaser*.

*“She [mother] just helps me.” *(Jill, 13-year-old female) Fifteen adolescents viewed the most influential role of the parent as that of *encourager. *The adolescents contributed their continued commitment and followup to treatment to the consistent encouragement that their parents provided them throughout treatment. Adolescents voiced a strong need for parental encouragement during treatment and discussed this particular role of the parent as essential to their continued stability:

*“She makes sure I get there [mental health facility].”* Several adolescents (*n* = 7) described that one manner in which their parents/guardians were influential in contributing to their stability was the role the parent played as a *transporter*. Although this role did not receive the emphasis of the *encourager *role, adolescents from both age groups identified this role as an essential component to their overall continuation in treatment.

*“She [mother] makes sure I take it [medication] everyday.”* (Angela, 15-year-old female) Adolescents also identified their parent as the *administrator *of their medications. Of the eleven adolescents currently receiving medication intervention, six discussed this particular role of their parent/guardian in terms of the significant influence on their continued stability. Further probes provided information on how the adolescent perceived this particular role. Among those taking medications, four viewed this role in a positive light, stating that the reminders from parents to take their medication(s) were helpful, while the remaining two viewed the parent's reminders as annoying.

*“She buys it for me.” *(John, 17-year-old male) Another influential parental role described by adolescents (*n* = 3) was that of a *purchaser. *The adolescents considered the task of their parents purchasing the medications as a positive influence on them remaining stable and continuing in treatment:

*She calls over here [mental health facility] and gets the prescription and then she buys it for me. If she did not do that I wouldn't have ‘em…that would not be a good thing for me, so, that's important for me to stay stable. *(John, 17-year-old male)

*“I encouraged myself.” *Seven adolescents (four 12 to 14 years old and three 15 to 17 years old) stated that in addition to their parents, their own influence led them to remain in treatment:

*I just wanted to stay in therapy because I think it helps me…so I guess you could say that I encourage myself because I see what good it does for me. *(Angela, 15-year-old female)

## 9. Others Who Influence

Two 12 to 14 years old and one 15 to 17 years old identified the nurse practitioner as influential in their remaining on medication(s) and two 12-to-14-year-old participants identified their therapist as influential in them remaining in therapy. Mentors were identified as influential in the adolescent remaining in treatment by three 12 to 14 years old, and two 15 to 17 year-olds identified a relative who was influential on them remaining in treatment.

## 10. Definition of “Consent to Treatment” 

Minor consent laws afford adolescents the right to seek and receive psychiatric mental health treatment without the permission of their parents. Given these adolescents have consented to medication therapy, psychotherapy, or both, exploring their understanding of consent to treatment is integral to understanding the decision-making process of adolescent consenting. Eleven participants (six 12 to 14 years old and five 15 to 17 years old) provided examples to explain their understanding of the minor consent laws.

*“You say you want to come to treatment.”* Of the eleven adolescents who provided a definition of “consent to treatment”, five provided examples that paralleled the technical definition of “consent to treatment”:

*It's like you allow…you say that you want to go to treatment and you're willing to go. *(John, 17-year-old male)

*That you say you want to come to treatment…that you say that you want to get help. *(Jill, 13-year-old female)

*It's like you get, you say “okay”, I will do it [receive treatment]…that it means I will do something or do it and I agree to it*.  (Dave, 13-year-old male)

Two adolescents provided a definition of “consent to treatment” that was partly correct. Based on the current North Carolina minor consent law, the sections identified by italic bold are not accurate:

*Well, it means that you have to be willing to have treatment, and no one can force you into doing anything…* **you have to have a parent or a guardian with you to sign the papers and everything**. (Barbara, 14-year-old female)

*Consent to treatment, is, um, like not only, um, not only affirmative, but just kind of like an agreement to, um, carry out all the, all of the components, like, well, the ask, well the assent of* **the, um, minor is just kind of agreeing with the consent [of the parent].  **(Tim, 16-year-old male)

*“Somebody can make you take it.”* The responses of the remaining four participants who provided a definition of “consent to treatment” spoke of this concept in terms of what was required or expected of them related to continued treatment, or what services they might receive at the facility:

*It means that like the doctor give you some medication and you have to take it…that you just do what they [doctor] tell you to do. *(Mack, 15-year-old male)

*Consent mean [sic] that somebody can make you take it [medication]. But parent consent means parent permission. *(Elaine, 17-year-old female)

*It means that I will get a mentor and sign up for anger management. *(Jill, 13-year-old female)

*Consent to me means that I will accept it [medication and/or therapy], that I will take whatever they [treatment facility] give me without any problem, that I won't make a big deal about it or fight about it…that I will come and whatever they suggest, I will agree to, like if they think it's best to have you put on medication, I'll take it. *(Lauren, 15-year-old female)

*“I do not know.”* When asked “what does *consent to treatment* mean to you?” five of the adolescent participants answered “I do not know.” Further probes included “what does it mean to you when you agree to treatment?”, or “what is involved in your agreeing to treatment?” These five respondents (three 12 to 14 years old and two 15 to17 years old) held to their original answer, “I do not know.” It was clear from this response that these participants either did not understand the question, or the adolescent was not aware that there was some level of required agreement on their part in consenting to treatment.

As indicated by these examples, younger and older adolescents were equally represented among those who understood and those who did not understand the concept of “consent to treatment.”

## 11. Evaluation of Minor Consent Laws

Adolescents were asked to describe their thoughts about a law that provides minors the right to consent to psychiatric mental health treatment without their parent's permission.

*“Kids should not be making those type of decisions.” *An overwhelming number of adolescent participants (*n* = 13) did not agree with a law that allows someone their age to consent to treatment without their parent's permission. The most common theme among these respondents (*n* = 11) was they did not possess the *confidence* in making healthcare decisions without their parent's input. In addition to lack of confidence, some adolescents (*n* = 5) discussed their opposition to the minor consent law solely on the *age* of the adolescent. For example, “older than 16 or 17” was a representative response among those who referred to age as a strict determinant of when a minor should be allowed to consent without their parent's required permission:

*Some kids should not be making those type of decisions without their parent…they just donot know what to ask about and they may end up not asking about something or telling the doctor something that's important. *(Vivian, 16-year-old female)

*Well, I think deciding about something like that should be a family thing…that's how we did it, so we decided as a family to do the treatment, to come here, so that's why I did it because it was a family thing. *(James, 12-year-old male)

*I donot think anyone younger than fifteen could decide on that stuff without their parent, not no 12 year or 13 year-old, for sure. I donot even know about a fifteen year-old, if they could do it [decide on treatment without parent]. Cause they still donot know what's good for them at that age [fifteen], and then maybe there's some 12 year-olds who think they know a lot but they be having babies and stuff… they donot know nothing, but I think maybe 16 year-olds could, maybe, it just depends on what they have to decide, maybe therapy, but I sure donot know about medicine. *(Elaine, 17-year-old female)

Our parents should let us pick what we want to be in, like group and stuff, but they (parents) should be, should know about my health, because they have to know about what's going on with me and my health, because if something goes bad and they do not know what's goin' on, then I could be in trouble. (Barbara, 14-year-old female)

Only one adolescent (17-year-old male) emphatically believed that adolescents should be allowed to consent to psychiatric mental health treatment without the required permission of parents, regardless of age, mental health diagnosis, or treatment involved. Two adolescents considered the *type of treatment* involved when expressing their perception of whether it was acceptable for an adolescent to consent to treatment without their parent's permission:

*I think it's okay with some things, like therapy maybe, it's okay with if they decide on their own, but they may not be in the position to do it on their own, like when medicines involved, then they cannot, I cannot see how they decide on that, that's something more serious, taking medicine, and they then the parent should decide, that's what I think. *(Elaine, 17-year-old female)

*Like medicine obviously, I think the parent should be there when deciding on that. If it were up to me I would have never taken the medicine, and I'd probably still be failing every class, or I'd probably be dead if it were up to me to not do the treatment.* (Lauren, 15-year-old female)

### 11.1. Voice in Treatment Decisions

Eleven adolescents (six 12 to 14 years old and five 15 to 17 years old) discussed that they should be allowed a voice in treatment decisions, but the inclusion of parents in the decision-making process was important to them:

*I think that would be good [a law that allows her to decide on her own healthcare without her parent], that's kind of good, because I get to make my own decisions, but I think asking my mom would help out a lot…asking her about the treatment first before I do it, that would be helpful. *(Jill, 13-year-old female)

*You know, we should be able to make our own decisions about our healthcare, but we do need our parents…they can help us out with that kind of stuff.* (Dave, 13-year-old male)

*It would be okay to have something like that [a law that allows her to decide on her own healthcare without her parent] cause I like to make my own decisions, but I donot think I could make those decisions without my mom, like treatment stuff. *(Jean, 12-year-old female)

*Yeah, I like that idea [a law that allows her to decide on her own healthcare without her parents], yeah the child should have some rights, but the child ain't so smart she know everything that the parent know…the parent, they look into things a little bit more then their child do. *(Vivian, 16-year-old female)

*I think maybe the kid should have opinions in the situation, like, if the child really thinks that he doesnot need whatever, then they should consider that. But, if they really need it, therapy and stuff, if they think they do not need therapy and they have scars going all up and down their arm and they're threatening suicide, then obviously they need some help, so I think the parent should definitely be involved when it comes to that type of stuff, especially with the medicine stuff. *(Lauren, 15-year-old female)

Further probing about inclusion of parents provided more detailed information about this aspect of consenting. One adolescent (17-year-old male) viewed requiring permission from the parent as unnecessary when it came to deciding on his psychiatric mental health treatment and that the input from his parents would most likely not make a difference in the decisions he made about treatment. One adolescent (12-year-old male) stated “I think it [treatment decisions] should be talked over with the kid and they decide about things with the parent.” Three adolescents (two 12 to 14 years old and one 15 to 17 years old) argued that the reason the current minor consent law should not exist is that the parent should be the one who decides on healthcare treatment and that the minor is not capable of making such decisions. When the term “capable” or similar terms were used by the adolescent, they were further asked to explain their meaning of “capable”. The adolescents described *capable* in terms of their inability to make decisions about issues that they had limited knowledge about, specifically related to choosing between different medications.

## 12. Discussion

The findings of this study suggest that younger and older adolescents are successful in completing the first two steps of the decision-making process (recognizing that a decision is required and understanding the goals). Adolescents in this study readily recognized that a decision from them about initial treatment and goals was necessary. However, it was typical for adolescents to complete the first two steps of the decision-making process in collaboration with their parents.

One consistent finding related to the first two steps of the decision-making process was that adolescents discussed the decisions they made and the desired goals of treatment in specific terms of their psychiatric mental health symptomatology, such as the desire to feel or act better now. For example, adolescents spoke of deciding on treatment and establishing short-term goals based on their desire to decrease sadness and tearfulness and increase their ability to focus and concentrate in school. Although, adolescents consistently made strong connections between what they were feeling or experiencing and the initial decisions made about treatment, the goals never moved beyond those of the short-term goals to feel better and act better. From a developmental perspective, this would be expected given that adolescents generally focus on the here and now when it comes to making decisions [4].

In relation to the first two steps of the decision-making process, the findings of this study are in contrast to what is observed in clinical settings [28]. In outpatient psychiatric mental health settings, younger adolescents seem to be less aware than older adolescents that a decision about treatment is needed. However, based on the findings of this study, younger and older adolescents equally recognize that decisions about their treatment are needed. One explanation for younger adolescents appearing to be less likely to recognize the requirement of a decision may be as simple as the clinician not consulting with the younger adolescent about this step, but with the parent instead. With older adolescents, clinicians may discuss initiation of treatment directly. However, when it comes to addressing the initial treatment decisions of the younger adolescent, the clinician may direct the discussion to the parents only, thus taking the younger adolescent out of the communication forum. This study provides greater insight into the ability of younger and older adolescents to recognize that treatment decisions are required, stating treatment goals, and verbalizing the details of these two decision-making steps in their own words.

Although younger and older adolescents readily identified the benefits of taking the medications and receiving psychotherapy, their ability to identify the risks and consequences related to these interventions was limited. The findings from this study are similar to researchers [7, 29] who examined risks identification of adolescents in healthcare situations. Adolescents in these studies did not readily identify risks of healthcare interventions, especially if the adolescent had not experienced previous side effects or adverse reactions to the intervention. From a cognitive developmental perspective, the expectation would be that younger adolescents would not think at a level other than immediate [4, 23]. Therefore, the findings of this study are in line with what others have reported in that younger adolescents experience difficulty in identifying the risks and consequences of treatment choices.

Identifying future consequences requires the adolescent to think in abstract terms, which is a defining characteristic of the formal operational stage of development [23]. Bloom [30] identifies six levels of learning. Within these levels, consideration is given to the concept of critical-thinking. It is important for the adolescent to develop critical-thinking skills in order to make decisions [23]. The development of critical thinking skills is dependent on the adolescent's ability to analyze, synthesize, and evaluate information [30]. For example, in the current study, in order to understand the future consequences of psychiatric mental health treatment choices and assimilate and integrate information about treatment options, the adolescent had to be able to analyze and synthesize the information presented. Based on the participants' ages (12 to 17 years old), it was expected that the older adolescents (15 to 17 year-olds) would respond more than younger adolescents (12 to 14 years old) to interview questions from a formal operational stage of development perspective, which includes the ability of the adolescents to synthesize and evaluate the initial treatment information presented to them [28].

Adolescents in this study did not recognize the future consequences of their treatment decisions. Most importantly, adolescents did not assimilate and integrate the information presented to them about their treatment options. These findings are similar to those of Lewis [29] and Urberg and Rosen [31] who reported that younger and older adolescents in their study were not capable of integrating information about the treatment interventions represented in their studies. The findings of this study are similar to the findings of others in that adolescents do not independently inquire about future implications of the treatment options. Further, adolescents in this study did not assimilate and integrate information unless this process was completed in collaboration with their parents. There was no evidence that adolescents in this study were functioning in the formal operational stage of development, nor were they using critical thinking skills (synthesis and evaluation) when it came to consenting to psychiatric mental health treatment.

## 13. Conclusions

In summary, findings of this study suggest that, unless completed in collaboration with their parents or legal guardians, 12-to-17-year-old adolescents do not identify consequences *(step 3)* and assimilate and integrate information *(step 4)* when it comes to deciding about psychiatric mental health treatments. These findings support the argument of those who oppose the expansion of the minor consent laws. However, the extraordinary experiences of adolescents gleaned from the current study provide support for those arguing in favor of minor consent laws. For example, the description of one particular adolescent's experiences reverberates. James is the 12-year-old male participant who was sexually molested by his mother and friends of his mother, for several years. James had a supportive and caring grandmother to disclose the details about the sexual abuse inflicted upon him. However, the possibility of James living with this abuse without a confidante is easily contemplated. Proponents of the current minor consent law would argue that James represents those for whom minor consent laws were intended. Specifically, proponents of minor consent laws would posit that adolescents like James benefit from these laws because they allow the adolescent to consent to psychiatric mental health treatment when it is the parent who is instigating the problem or substantially contributing to the adolescent's mental health issues. In the case of parental abuse, it is unlikely that the parent would agree to the adolescent receiving psychiatric mental health treatment. Current minor consent laws would allow the adolescent to seek treatment without the parent knowing, which is perhaps the only way that some adolescents would seek refuge when it is the behaviors of the parents that are contributing to their mental illness. The overall findings of this study support those who oppose the current minor consent laws. However, based on the experience of James, which is representative of many adolescents, the negative implications of changing this law cannot be overlooked.

Finally, the findings of this study indicate that knowledge and understanding of the minor consent laws by adolescents is significantly limited. Most adolescents in this study were not aware of an existing law that allows minors to consent to psychiatric mental health treatment without their parent's permission. Considering the findings of this study, there is a need for increased dialogue among adolescents, parents, healthcare professionals, and legislators related to this law. This dialogue should focus on three main topics. First, for clinical and legal reasons, societal awareness of minor consent laws should be increased. If increased awareness leads to more adolescents seeking treatment without their parents, then further research is warranted in order to thoroughly evaluate the outcomes of the law. Second, if further research supports the findings of this study, that adolescents make their best decisions in psychiatric mental health settings when collaborating with parents, then minor consent laws should be considered for amendment to better reflect the decision-making process and cognitive development of adolescents. Third, consideration should be given to the voice of the parents. Perhaps a change in the law would not only reflect any concerns expressed by parents, but also provide clear legal and clinical guidelines related to adolescents consenting to psychiatric mental health treatment.

## Figures and Tables

**Table 1:** Gender and race frequency distribution by age group.

Age group	Caucasian (*n* = 6)		African-American (*n* = 10)	
Total (*n*)	Female (*n* = 1)	Male (*n* = 5)	Female (*n* = 8)	Male (*n* = 2)
12 to 14 (9)	0	3	5	1
15 to 17 (7)	1	2	3	1

**Table 2:** Diagnosis and frequency by age group.

Diagnosis	12 to 14 years old	15 to 17 years old
	(*n* = 9)	(*n* = 7)
ADHD	3	1
ODD		2
MDD		2
PTSD	1	
Social phobia	1	
ADHD/DBD	2	
ADHD/ODD	1	
ADHD/MDD		2
ADHD/PTSD/ODD	1	

Note: ADHD: attention deficit hyperactivity disorder; ODD: oppositional defiant disorder; MDD: major depressive disorder; PTSD: post traumatic stress disorder; DBD: disruptive behavior disorder.

**Table 3:** Frequency of current management regimen by age group.

Age group Total (*n*)	Medication only (*n* = 4)	Therapy only (*n* = 6)	Combined (*n* = 6)
12 to 14 (9)	2	3	4
15 to 17 (7)	3	2	2

**Table 4:** Participant grade level progression frequency by age group.

Age group total (*n*)	On time grade	1-year behind grade	≥2-years behind grade
12 to 14 (9)	4	4	1
15 to 17 (7)	2	4	1
